# Diagnostic performance of artificial intelligence in periapical radiography: a systematic review

**DOI:** 10.1007/s10266-026-01401-8

**Published:** 2026-05-13

**Authors:** Tulin Tasdemir, Melek Tassoker, Abdullah Arpacı, Ayşenur Kula, Ferhat Enes Kocabiyik, Mehmet Enes İyişenyürek, Huseyin Hakli

**Affiliations:** 1 Faculty of Dentistry, Department of Pediatric Dentistry, Nigde Omer Halisdemir University, 51240 Nigde, Türkiye; 2 Faculty of Dentistry, Department of Dentomaxillofacial Radiology, Necmettin Erbakan University, 42050 Meram, Konya Türkiye; 3 Department of Computer Engineering, Necmettin Erbakan University, 42090 Konya, Türkiye

**Keywords:** Artificial intelligence, Deep learning, Dentistry, Diagnostic performance, Periapical radiography

## Abstract

To systematically evaluate the diagnostic accuracy of artificial intelligence (AI) models in periapical radiography for detection, classification, and segmentation tasks compared to human experts, while critically appraising methodological quality and risk of bias. The systematic review was conducted in accordance with PRISMA guidelines and registered in PROSPERO (CRD420251132033). Strict exclusion criteria were applied to studies with insufficient dataset sizes (< 200 images) lacking augmentation, ambiguous reference standards, or reporting only accuracy without complementary metrics. A comprehensive literature search was conducted on June 23, 2025, across five electronic databases: PubMed/MEDLINE, Scopus, ScienceDirect, Web of Science, and IEEE Xplore. The search strategy combined keywords related to “periapical radiography” (e.g., periapical X-ray), “artificial intelligence” (e.g., deep learning, neural networks), and specific diagnostic tasks, with no restrictions on publication date or language. Studies utilizing artificial intelligence models for diagnostic tasks on periapical radiographs and validating performance against a human reference standard (expert consensus) were eligible for inclusion. Out of 544 identified records, 47 studies met the full eligibility criteria and were included in the qualitative synthesis. AI models possess a high diagnostic potential in periapical radiography, performing at a level comparable to human experts in tasks such as pathology detection, anatomical segmentation, and implant classification. However, their clinical applicability is currently limited by a high risk of bias, lack of external validation, reliance on cropped datasets, and the use of human consensus as a surrogate reference standard. Future research must prioritize full-arch evaluations, anatomical region-specific performance reporting, and adherence to AI-specific standardized metrics.

**Supplementary Information:**

The online version contains supplementary material available at https://doi.org/10.1007/s10266-026-01401-8.

## Introduction

Intraoral periapical radiography is a cornerstone imaging modality in endodontics and restorative dentistry because it provides high-resolution visualization of the tooth, periapical region, periodontal structures, restorations, and common treatment-related findings. Despite its routine use, interpretation of periapical radiographs remains challenging: subtle periapical radiolucencies may be obscured by anatomic superimposition, early disease may not yet be radiographically apparent, and diagnostic decisions can vary with reader experience and workload. These limitations motivate the development of computer-aided approaches that can support clinicians in detection and decision-making while maintaining efficiency in daily practice [1, 2].

Recent advances in artificial intelligence (AI), particularly deep learning using convolutional neural networks, have accelerated research on automated analysis of dental radiographs. In periapical radiography, AI systems have been investigated for tasks including detection of periapical radiolucent lesions/apical periodontitis [3], marginal bone loss, caries [4], restorations, and endodontic treatment related features [5, 6]. Primary diagnostic accuracy studies generally report promising performance for several clinically relevant findings in periapical radiographs and suggest that AI may function as an assistive “second reader” in some scenarios [7].

However, reported performance varies substantially across studies due to heterogeneity in datasets, reference standards (e.g., expert consensus versus additional imaging), disease prevalence, annotation protocols, and the specific AI task formulation (classification, detection, or segmentation). Methodological concerns such as limited external validation, potential spectrum bias, and inconsistent reporting can also affect the trustworthiness and clinical generalizability of results. Systematic synthesis is therefore needed to clarify the overall diagnostic performance of AI in periapical radiography and to identify which clinical indications and study designs provide the most reliable evidence [8].

Accordingly, this systematic review aims to evaluate and summarize the diagnostic performance of AI systems applied to periapical radiography, with a focus on clinically relevant outcomes (e.g., F1 score, recall, accuracy, AUC) and study characteristics that influence performance (e.g., dataset size, reference standard, validation strategy, and risk of bias). By consolidating the available evidence, we seek to inform clinicians, researchers, and developers about the current capabilities and limitations of AI in periapical radiographic diagnosis and to highlight priorities for future prospective and externally validated studies.

## Methods and materials

This diagnostic accuracy systematic review was conducted following the Preferred Reporting Items for Systematic Reviews and Meta- Analyses (PRISMA) guidelines [9] and the completed PRISMA 2020 reporting checklist. The review protocol was registered and allocated with the CRD420251132033 in the PROSPERO.

### Eligibility criteria

The objective of this systematic review was to evaluate the diagnostic performance of artificial intelligence models (**Index Test**) applied to periapical radiographs (**Population**) for detecting, classifying, or segmenting dental structures and pathologies. The diagnostic accuracy of these models was compared against expert human interpretation or clinical consensus (**Reference Standard**). The primary outcomes of interest (**Outcome**) included Accuracy, Sensitivity, Specificity, Precision, Recall, and F1 Score, while secondary metrics such as the Dice Coefficient and Area Under the Curve (AUC) were recorded where reported.

Studies were left out if they do not report detailed training/testing data, do not clearly outline the AI methods, or are themselves reviews. Also, studies using imaging modalities other than periapical radiographs or not addressing the predefined outcomes, along with conference abstracts lacking enough data for extraction, were excluded.

### Information source and search

A comprehensive literature search was conducted on June 23, 2025, across electronic databases including PubMed/MEDLINE, Scopus, ScienceDirect, and Web of Science. No restrictions were placed on the publication date to ensure all relevant studies were identified. The search strategy utilized the following keywords and MeSH terms: (“periapical”) AND (“artificial intelligence” OR “deep learning” OR “neural network”) NOT (“panoramic” OR “CBCT”) (Table 1).

**Table 1:** Search strategy, keywords, and results in electronic databases

Database	Search Query/Syntax	Results (*n*)
PubMed/MEDLINE	(“periapical“[All Fields]) AND (“artificial intelligence“[MeSH Terms] OR “artificial intelligence“[All Fields] OR “deep learning“[All Fields] OR “neural network“[All Fields]) NOT (“panoramic“[All Fields] OR “CBCT“[All Fields])	**131**
Scopus	TITLE-ABS-KEY (“periapical”) AND TITLE-ABS-KEY (“artificial intelligence” OR “deep learning” OR “neural network”) AND NOT TITLE-ABS-KEY (“panoramic” OR “CBCT”)	**204**
Web of Science	TS=(“periapical”) AND TS= (“artificial intelligence” OR “deep learning” OR “neural network”) NOT TS= (“panoramic” OR “CBCT”)	**163**
ScienceDirect	(“periapical”) AND (“artificial intelligence” OR “deep learning” OR “neural network”) AND NOT (“panoramic” OR “CBCT”)	**171**
IEEE Xplore	(“periapical”) AND (“artificial intelligence” OR “deep learning” OR “neural network”)	**19**

### Study selection

After removing duplicate articles, articles irrelevant to the topic were eliminated by three independent researchers (T.T., M.T., H.H.). During the second screening phase, the title, abstract, and subsequently the full text of potentially relevant studies were obtained for further evaluation, and any disagreements were resolved by consensus. Inter-rater reliability for study selection was assessed using Cohen’s kappa coefficient, yielding a score of 0.92, indicating excellent agreement between reviewers.

### Data collection and extraction

Four different reviewers individually collected data from each of the studies included in the present systematic review (A.A., A.K., F.E.K., M.E.İ.). Two different reviewers revised the data extraction to resolve discrepancies and disagreements (T.T., M.T., H.H.). The extracted data items were: bibliographic information (author’s name, publication year), aims of the study, dataset size (train, validation, test if mentioned in the study), deep learning approach (i.e., classification, regression), data pre-processing and augmentation (artificially increasing the size of a dataset by creating modified versions of existing data points), deep learning model architecture, the specific reference standard utilized to establish the ground truth (e.g., single expert annotation, multi-expert consensus, or histological validation), the reported diagnostic performance metrics, and the corresponding results.

While Deep Learning (DL) models were the primary focus, studies utilizing traditional machine learning algorithms (e.g., SVM, Random Forest) requiring manual feature extraction were excluded to maintain methodological homogeneity. A rigorous assessment of dataset characteristics was performed to mitigate the risk of overfitting and low generalizability. Studies were excluded based on the following hierarchical criteria:

1. **Small datasets without augmentation**: Studies with small-to-medium datasets (< 1,000 images) that failed to report any data augmentation techniques (e.g., rotation, flipping, mosaic) were excluded due to the high risk of overfitting [10]. However, studies with limited datasets were exceptionally included if they addressed niche pediatric topics (e.g., primary dentition) and employed robust augmentation strategies to compensate for data scarcity.
2. **Insufficient sample size**: Studies trained on extremely small datasets (< 200 images) were excluded based on statistical power considerations. Methodological guidelines emphasize that splitting small datasets for validation poses a severe risk of unstable performance estimates and lack of external validity [11, 12]. To ensure a statistically reliable evaluation, we established a minimum threshold of 200 images. This cutoff ensures that, assuming a standard 20% train-test split, the test set comprises at least 40 images, which is the approximate minimum required to achieve a normal distribution and reliable confidence intervals according to the Central Limit Theorem [13].
3. **Absence of augmentation in medium datasets**: For studies with medium-sized datasets (1,000–2,000 images), inclusion was contingent upon the complexity of the task. Complex tasks (e.g., caries detection) required augmentation to prevent overfitting, as standard deep learning models typically demand large-scale data to capture subtle pathological features [14]. Conversely, anatomically standardized tasks (e.g., tooth numbering) with sufficient data volume were accepted without explicit augmentation. Studies with large-scale datasets (> 2,000 images) were included regardless of augmentation, as the substantial data volume itself provides sufficient variance to represent the target population diversity, reducing the dependency on synthetic data generation [15, 16].
4. **Methodological flaws**: Studies lacking a clearly defined test set (data leakage risk) or failing to define a clear reference standard (ground truth) were excluded, as these are fundamental requirements for reproducible AI research defined in the Checklist for Artificial Intelligence in Medical Imaging (CLAIM) checklist [17]. Furthermore, studies reporting only ‘Accuracy’ without complementary metrics (e.g., Sensitivity, F1-Score) were excluded, as accuracy alone can be mathematically misleading in unbalanced medical datasets [18].

Due to the heterogeneity of AI tasks (e.g., classification vs. segmentation), the reported performance metrics varied across studies. Therefore, all available metrics validated against the reference standard were extracted to provide a comprehensive overview. A quantitative meta-analysis was not feasible due to the significant heterogeneity observed across the included studies. This heterogeneity stemmed from three main factors: (1) Variability in AI Tasks: The studies ranged from classification and object detection to semantic segmentation, necessitating different performance metrics (e.g., Accuracy/Sensitivity vs. Intersection over Union [IoU]/Dice Coefficient). (2) Dataset Diversity: There were substantial differences in dataset sizes, imaging protocols, and ground truth labeling methods (e.g., bounding box vs. pixel-level mask). (3) Metric Inconsistency: Not all studies reported a standard set of metrics; while some prioritized Sensitivity/Specificity, others focused on Mean Average Precision (mAP).

Therefore, a structured narrative synthesis was adopted. Extracted data were tabulated and standardized where possible (e.g., converting all probability values to percentages). To facilitate a meaningful comparison, the studies were clustered into five clinical domains: (i) Restorative, (ii) Endodontic, (iii) Periodontal/Implant, (iv) Anatomical/Workflow, and (v) Multitask Applications. Performance metrics were reported descriptively for the best-performing model in each study.

### Risk of bias and applicability

Two reviewers (T.T. and M.T.) independently evaluated the risk of bias and applicability concerns of the included studies using the QUADAS-2 tool. Discrepancies were resolved by consensus, and a third reviewer (H.H.) was consulted when agreement could not be reached.

## Results

### Study selection

**Fig. 1:**
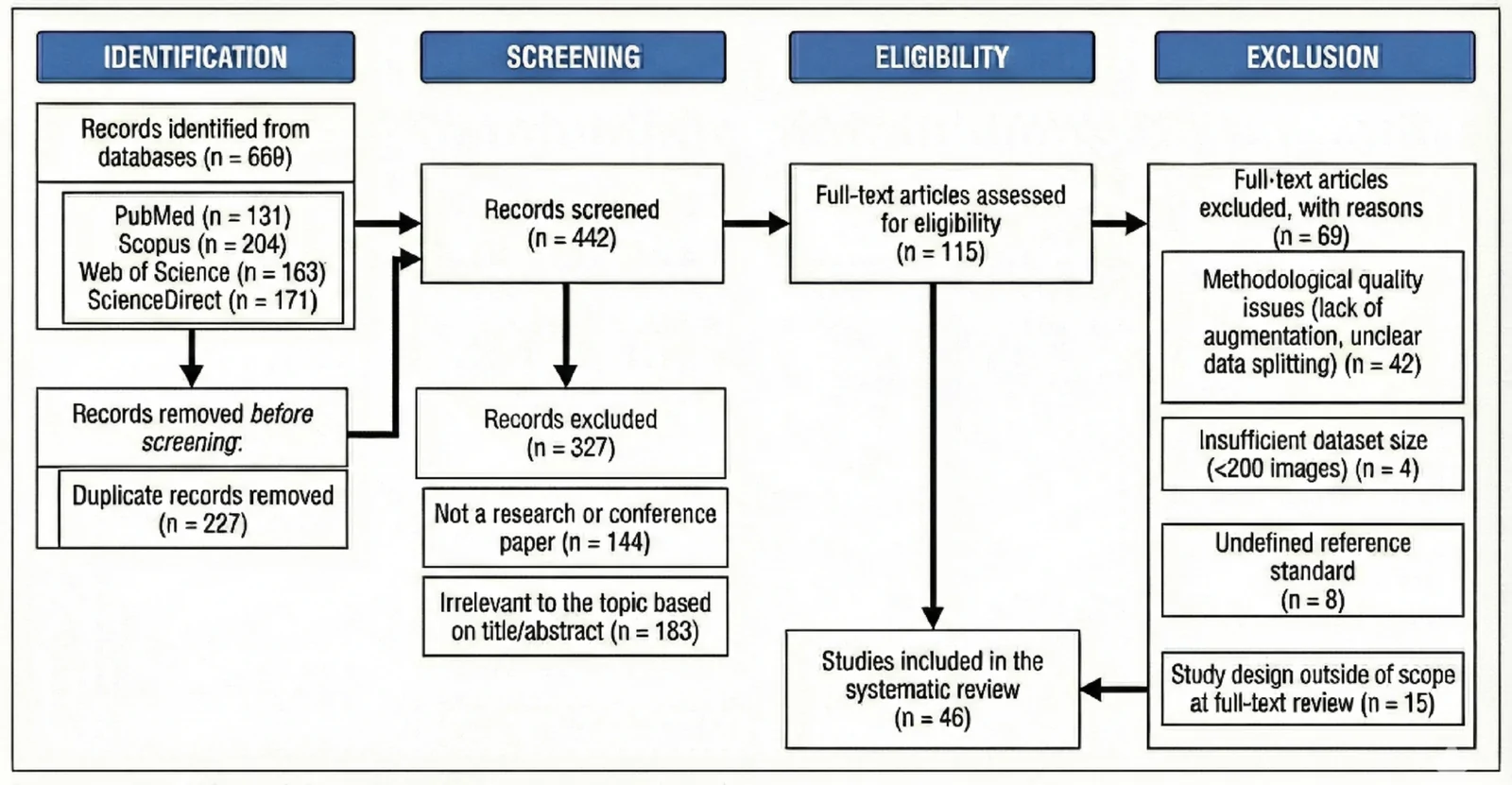
PRISMA flow diagram for study selection

The initial database search yielded a total of 544 records. After removing duplicates, 298 titles and abstracts were screened. Of these, 115 potentially relevant articles were retrieved for full-text evaluation. Following the rigorous eligibility criteria detailed in the methodology—specifically regarding dataset size, augmentation necessity, and methodological reporting standards—68 studies were excluded. The primary reasons for exclusion at this stage were insufficient sample size (< 200 images) without statistical validation, lack of essential performance metrics beyond accuracy, and absence of data augmentation in small-scale datasets. Ultimately, 47 studies met all inclusion criteria and were included in the qualitative synthesis. The complete selection process is illustrated in the PRISMA flowchart (Fig. 1).

### Study characteristics

A total of 47 studies met the strict inclusion criteria and were included in this systematic review. With respect to publication year, the earliest included study dates back to 2018, with publications spanning the period from 2018 to 2025. The chronological distribution of the selected articles indicates a significant surge in research activity in recent years, with the vast majority being published between 2023 and 2025. This trend underscores the growing integration of deep learning technologies into periapical radiography workflows.

The included studies were stratified into five distinct clinical domains based on their primary diagnostic tasks:

- **Restorative dentistry and caries detection (n** = 11): Research in this domain concentrated on the classification and segmentation of dental caries, as well as the detection of pulp exposure and restorative materials.
- **Endodontics (n = 12)**: These studies primarily addressed the detection of periapical lesions, vertical root fractures, and the assessment of root canal treatment quality or broken instruments.
- **Periodontology and implantology (n = 15)**: This category represented the largest group, focusing on alveolar bone loss detection, periodontal staging, and implant classification/segmentation.
- **Anatomical detection and workflow (n = 5)**: This group included studies aiming at automated tooth numbering, anatomical landmark identification, and radiograph assembly.
- **Multitask AI models (n = 4)**: A smaller subset of recent studies proposed comprehensive models capable of performing simultaneous tasks, such as detecting caries and periodontal bone loss concurrently.

Regarding the technical characteristics, a diverse range of Deep Learning (DL) architectures was utilized. For classification tasks, Convolutional Neural Networks (CNNs) such as ResNet, VGG, and EfficientNet were frequently employed. For object detection, the YOLO (You Only Look Once) family (v3 to v8) and Faster R-CNN were the predominant architectures. Segmentation tasks relied heavily on U-Net and its variants (e.g., ResUNet, Mask R-CNN).

Dataset sizes exhibited substantial heterogeneity, ranging from small datasets (< 300 images) to large-scale repositories (> 10,000 images). Consistent with the inclusion criteria, data augmentation techniques—including rotation, flipping, cropping, and mosaic—were universally applied, particularly in studies with limited initial data, to prevent overfitting and enhance model generalization. The performance of these models was evaluated using standard metrics, with Accuracy, Sensitivity (Recall), Precision, F1-Score, and Intersection over Union (IoU) being the most commonly reported outcomes. Detailed characteristics of the included studies are summarized in Table 2.

**Table 2:** Characteristics of the included studies (*n* = 47)

Author (Year)	Clinical task (Annotation)	Dataset (initial and final) and augmentation	AI model	Key results*
**I. Restorative dentistry & caries detection**				
*Baquhaizel et al. (2023)* [19]	Caries Classification (Class Label)	Init: 639 Final: 2532 (Zoom, Shift)	VGG16	Acc: 99% Sens: 99% Spec: 98% F1: 99%
*Yavsan et al. (2025)* [20]	Pediatric Approximal Caries (Bounding Box)	Init: NA Final: 415 (Mosaic, Color)	YOLOv8	mAP: 0.90 F1: 90% Prec: 91% Recall: 89%
*Choi et al. (2018)* [21]	Caries Segmentation (Mask)	Init: 475 (Rotation, Flip)	Custom CNN	F1 Score: 74% *(Only F1 reported)*
*Zheng et al. (2021)* [22]	Caries Depth Classification (Class Label)	Init: 844 (Rotation, Shift)	ResNet-18	Acc: 86% Sens: 89% Spec: 86% Prec: 85%
*Zhu et al. (2022)* [23]	Caries Detection (Bounding Box)	Init: 200 Final: 800 (Flip, Contrast)	Faster R-CNN	F1 Score: 68% *(Only F1 reported)*
*Altukroni et al. (2023)* [24]	Pulp Exposure Detection (Bounding Box)	Init: 3445 (Mosaic, Hist Eq)	YOLOv5	mAP: 0.95 F1: 92% Prec: 92% Recall: 91%
*Liu et al. (2024)* [25]	Caries Classification (ROI Crop)	Init: 12,524 (Flip, Rotation)	ResNet + SAM	Acc: 88.5% Spec: 88.7% AUC: 0.95
*Khan et al. (2025)* [26]	Caries Segmentation (Mask)	Init: 17,840 Final: 19,046 (Flip, Rot)	ResUNet	Acc: 98% IoU: 0.60
*Priya et al. (2024)* [27]	Caries Classification (Class Label)	Init: 300 Final: 1800 (Flip, Rot)	ResNet50 (Fusion)	Acc: 95.5% Prec: 94.6%, Recall: 95.2% F1: 94%
*Ying et al. (2024)* [28]	Caries Detection (Bounding Box)	Init: 392 Final: 1730 (Flip, Rot)	YOLOv5	Prec: 93% Spec: 94% F1: 87% Recall: 82%
*Liu et al. (2025)* [29]	Caries Segmentation (Mask)	Init: 5,569 (No Augmentation)	YOLOX + FCN	Acc: 82% DSC: 0.84 Prec: 83% Recall: 85%
**II. Endodontic applications**				
*Xu et al. (2022)* [30]	Root Canal History (ROI Crop)	Init: 230 Final: 920 (Flip, Contrast)	VGG16 + SVM	Acc: 97.2% Recall: 94.6% Spec: 100%
*Chuo et al. (2022)* [31]	Apical Lesion Classification (Class Label)	Init: 490 Final: 760 (Flip, Crop)	ResNet-50	Acc: 96.2% Sens: 98.5% Spec: 93.9%
*Ma et al. (2025)* [32]	Pediatric Pulpitis Classification (Polygon)	Init: 348 (Flip, Brightness)	EfficientNet-B2	Acc: 90.2% Sens: 76.9% Spec: 94.7% F1: 80%
*Pauwels et al. (2021)* [33]	Periapical Lesion Detection (Class Label)	Init: 280 Final: 5600 (Noise, Affine)	Custom CNN	Acc: 100% Recall: 100%
*Moidu et al. (2022)* [34]	PAI Scoring (Bounding Box)	Init: 3540 Final: 50,990 (Flip, Shear)	YOLOv3	Acc: 86% Recall: 89% Prec: 90% F1: 89%
*Liu et al. (2025)* [35]	Periapical Lesion Detection (ROI Crop)	Init: 1305 Final: 3393 (Flip, Sharpness)	ConvNeXt	Acc: 91.2% Recall: 98.5% Prec: 85.9% F1: 0.92
*Çetinkaya et al. (2025)* [36]	Broken Instrument/FEI (Bounding Box)	Init: 1050 (Flip, Noise)	YOLOv8	mAP: 0.98 Acc: 97.4% IoU: 1.00
*Çatmabacak and Çetinkaya (2025)* [37]	Broken Instrument (Polygon)	Init: 700 (Rotation, Flip)	DenseNet201	Acc: 90.5% AUC: 0.90 MCC: 0.81
*Ozsari et al. (2025)* [38]	Vertical Root Fracture (Class Label)	Init: 378 (Rotation, Flip)	DenseNet	Acc: 80% Prec: 81% Recall: 80% F1: 80%
*Jin et al. (2025)* [39]	Root Canal Filling Quality (Bounding Box)	Init: 1008 (Mosaic, Flip)	YOLOv5	Recall: 99.5% Prec: 85.6% F1: 92%
*Dennis et al. (2024)* [40]	Root Canal Treatment (Polygon)	Init: 1080 (Keras Augmentation)	Mask R-CNN	mAP: 88% Prec: 92% Recall: 92% F1: 90%
*Karkehabadi et al. (2024)* [41]	Endodontic Difficulty (Class Label)	Init: 1386 (Flip, Rotation)	VGG16	Acc: 87.6% Prec: 88.9%, Recall: 87.6% F1: 87.8%
**III. Periodontology and implantology**				
*Chen et al. (2023)* [42]	Peri-implantitis Severity (ROI Class Label)	Init: 456 Final: 1384 (Flip, Hist Eq)	YOLOv2	Acc: 90.4% Sens: 91.4% Spec: 89.3% Prec: 90.7%
*Santos et al. (2021)* [43]	Implant Marker Detection (Class Label)	Init: 1800 Final: 402,000 (Blur, Rot)	Custom CNN	Acc: 85% Recall: 89% Spec: 82%
*Kabir et al. (2021)* [44]	Perio Staging & Bone Loss (Mask)	Init: 700 (Crop, Flip)	Segmentation Net	AUC: 0.96 Dice: 0.96 IoU: 0.93
*Vera et al. (2023)* [45]	Peri-implant Bone Loss (Bounding Box)	Init: 2336 Final: 2920 (Rotation)	YOLOv3	mAP: 0.53-0.89
*Park et al. (2023)* [46]	Implant Size Classification (Polygon)	Init: 1320 Final: 15,540 (Flip)	VGG16	Acc: >99% F1: >97% Spec: >99%
*Chen et al. (2024)* [47]	Periodontal Bone Loss (Mask)	Init: 336 (Flip, Shift)	Mask R-CNN	Acc: 72.8% F1: 84.1% Recall: 97%
*Dai et al. (2024)* [48]	Periodontitis Staging (Class Label)	Init: 11,120 (No Augmentation)	AlexNet	Overall Acc: 80.2% Control Acc: 96.8%
*Yavuz et al. (2024)* [49]	Perio Health Classification (Class Label)	Init: 2618 (No Augmentation)	YOLOv8-cls	Acc: 77% F1: 78.2% Prec: 74.3% Recall: 82.4%
*Dujic et al. (2023)* [50]	Periodontal Bone Loss (Class Label)	Init: 21,819 (No Augmentation)	Vision Transformer	Acc: 85.2% AUC: 0.91 Recall: 89.8%
*Vilkomir et al. (2024)* [51]	Furcation Involvement (Class Label)	Init: 1078 (Flip, Scale)	ResNet-18	Acc: 96.4% Spec: 98% Prec: 97%
*Lin et al. (2024)* [52]	Bone Loss & CEJ Level (Polygon)	Init: 281 Final: 675 (Flip, Rot)	YOLOv8	Acc: 97% mAP: 98.9% F1: 97%
*Adnan et al. (2024)* [53]	Implant Segmentation (Mask)	Init: 300 Final: 1294 (Flip, Rot)	U-Net	Acc: 93.8% IoU: 86.4% Prec: 90%
*Fasih et al. (2025)* [54]	Implant Misfit (Polygon)	Init: 638 (Flip, Brightness)	ResNet-50	Acc: 92.7% Dice: 88.9%, Recall: 90.3%
*Lin et al. (2025)* [55]	Implant Brand Detection (Bounding Box)	Init: 241 Final: 884 (Mixup, Mosaic)	YOLOv10	Acc: 94.5% mAP: 99.2%, Recall: 93.3%
*Şahin and Eninanç (2025)* [56]	Lamina Dura Loss (Class Label)	Init: 701 (Flip, Shift)	ResNet (Custom)	Acc: 71.8% F1: 73% Sens: 73% Spec: 70.6%
**IV. Anatomical detection and workflow**				
*Görürgöz et al. (2022)* [57]	Tooth Numbering (Bounding Box)	Init: 1686 (No Augmentation)	Faster R-CNN	Recall: 98.6% F1: 87.2% Prec: 78%
*Kyventidis and Angelopoulos (2021)* [58]	Anatomical Region Class. (Class Label)	Init: 15,515 (Shift, Shear)	InceptionResNet	Acc: 89% F1: 86% Recall: 88%
*Mori et al. (2022)* [59]	Maxiller Canine Position Quality (Mask)	Init: 500 Final: 850 (Flip)	U-Net + AlexNet	Acc: 87.5% Recall: 92.5% Spec: 82.5%
*Pfänder et al. (2023)* [60]	Radiograph Assembly (Bounding Box)	Init: 4707 (Blur, Contrast)	ResNet-152	Acc: 99% F1: 79% Prec: 79%
*Ayyıldız et al. (2024)* [61]	Tooth Detection (Polygon)	Init: 6446 (Mosaic, Shift)	YOLOv5	Acc: 94.4% F1: 97.1% Recall: 98.8%
**V. Multitask AI models**				
*Chen et al. (2023) *[62]	Perio + Caries Simultaneous (B. Box)	Init: 11,000 (Rotation, Flip)	EfficientNet	Acc (Caries): 94.9% Acc (Perio): 95.4%
*Khan (2021)* [63]	Caries + Bone Loss (Mask)	Init: 206 (Elastic Transform)	U-Net	Dice: 98% mIoU: 97%
*Chen et al. (2023)* [64]	Tooth Pos + Bone Level (Mask)	Init: 8000 (No Augmentation)	Ensemble CNN	Overall Acc: ~90% Bone Level Acc: 92.6%
*Liu et al. (2024)* [3]	Tooth Seg + Lesion Detect (B. Box)	Init: 1305 Final: 3132 (Flip)	YOLOv5 + ConvNeXt	Acc: 90.9% mAP: 99.1% F1: 91.6%

*For studies evaluating multiple models or tooth types, the highest performance metrics are presented.

In accordance with our predefined eligibility criteria, the reference standard (gold standard) used to determine diagnostic accuracy in all 47 included studies was human expert annotation or clinical consensus. However, the methodological rigor of this human-derived ground truth varied considerably across the literature. While some studies employed a robust consensus mechanism utilizing a panel of multiple specialists (e.g., endodontists or periodontists) with calculated inter-rater reliability, a significant portion of the studies relied on the visual assessment of a single observer without reported intra-examiner calibration. Absolute clinical ground truths—such as histological validation for periapical lesions, 3D imaging (CBCT) verification for alveolar bone loss, or direct surgical exploration for vertical root fractures—were notably absent from the training and testing phases of these models, primarily due to the retrospective nature of the utilized radiographic datasets.

### Risk of bias and applicability

**Fig. 2:**
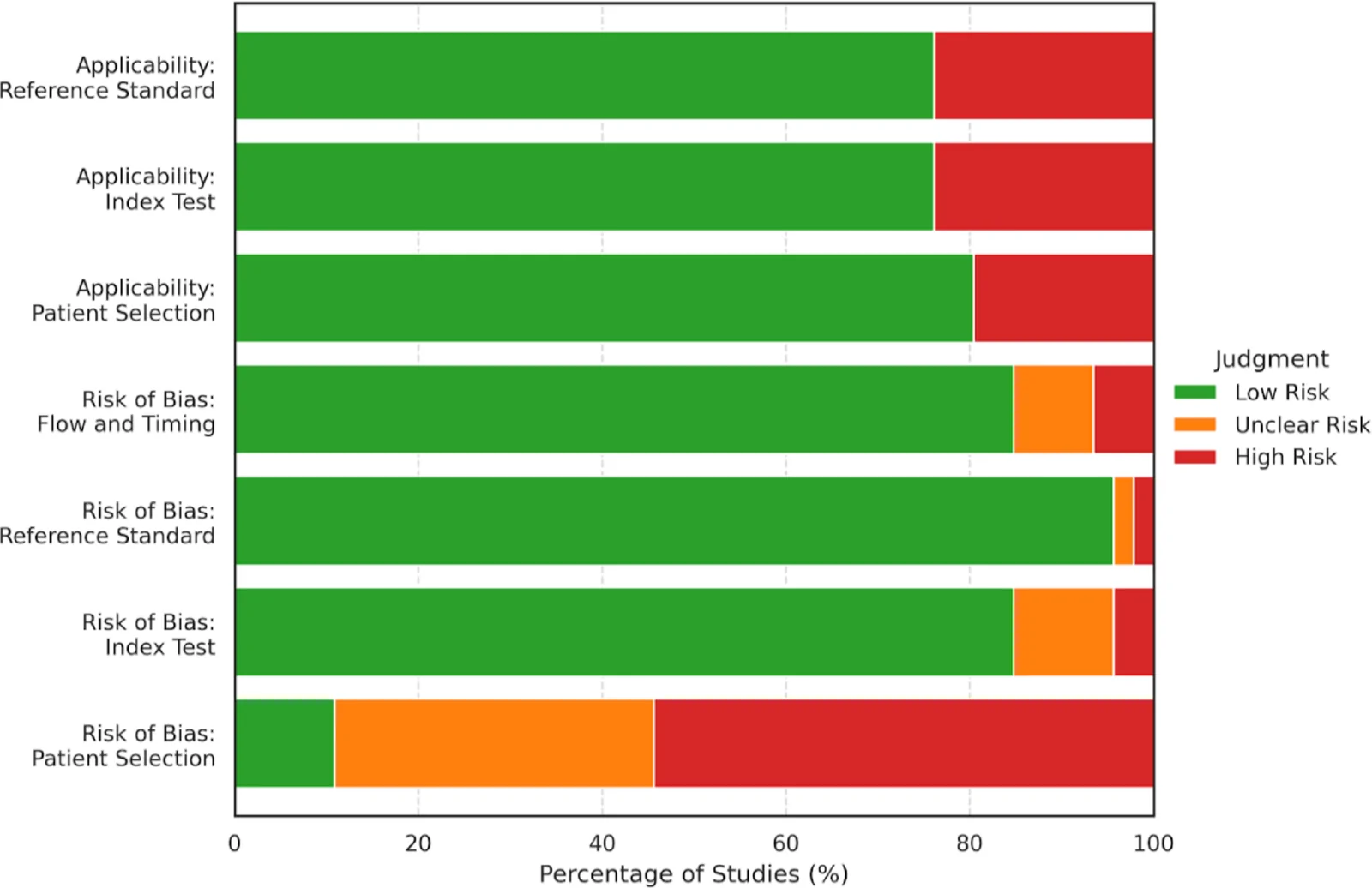
Risk of bias and applicability concerns according to the QUADAS-2 tool

The methodological quality of the 47 included studies was rigorously evaluated using the QUADAS-2 tool, with detailed assessments of risk of bias and applicability concerns stratified by clinical domain presented in Table 3. Overall, the critical appraisal revealed a predominant high risk of bias across the included literature, as summarized in Fig. 2. Among the 47 studies, only four studies (8.7%) [39, 46, 55, 60] demonstrated a “Low” risk of bias across all four domains. Conversely, a significant portion of the studies exhibited high risk, particularly in the “Patient Selection” and “Flow and Timing” domains, raising concerns about the potential overestimation of diagnostic accuracy in controlled settings compared to real-world clinical scenarios.

In terms of risk of bias, the “Patient Selection” domain was the most frequent source of methodological concern. A substantial number of studies, particularly in the fields of Restorative Dentistry and Endodontics, utilized pre-selected, non-consecutive datasets—often retrospective databases enriched with “perfect” examples of specific pathologies—rather than a random or consecutive sample of patients. This introduces a significant selection bias, as the spectrum of disease in these datasets may not reflect the complexity and variability encountered in routine clinical practice. Furthermore, the “Reference Standard” domain showed high risk in studies where the ground truth was established by a single observer without a consensus mechanism or intra-examiner calibration [25, 30]. However, studies involving multiple experts or histological validation were rated as low risk. The “Index Test” domain was generally robust, with most studies employing separate test sets (hold-out or cross-validation) to ensure blinded evaluation. Nevertheless, the “Flow and Timing” domain remained unclear or high risk in several studies due to unspecified intervals between the index test and reference standard, or failure to account for all patients in the final analysis.

Regarding applicability concerns, the “Patient Selection” domain again raised the most significant issues. Several studies, particularly in Periodontology and Endodontics, trained models on cropped Regions of Interest rather than full periapical radiographs. While this approach simplifies the computational task, it limits the direct clinical translation of the models, as automated detection systems in practice must handle full-sized images with anatomical noise. Additionally, the use of synthetic or augmented data in some studies further diverged from clinical reality. In contrast, the “Index Test” and “Reference Standard” domains generally demonstrated low applicability concerns, as the AI tasks (e.g., caries detection, alveolar bone loss assessment) and reference methods (expert consensus) were directly relevant to the review question.

A subgroup analysis by clinical domain revealed distinct patterns in methodological quality. Restorative Dentistry studies frequently showed high bias in patient selection, often relying on datasets specifically enriched with carious lesions. Endodontics and Periodontology studies generally reported high diagnostic accuracy, but methodological reporting flaws, such as unclear blinding of annotators, contributed to an overall unclear or high risk of bias rating. Notably, recent Multitask and Anatomical Models [3, 62] demonstrated better adherence to reporting standards, reflecting the maturation of the field, though challenges in explaining the “Flow and Timing” for complex, simultaneous tasks remained.

**Table 3:** Detailed risk of bias and applicability concerns summary for included studies according to the QUADAS-2 tool

Study ID	Risk of bias domains (*P*/I/*R*/F)	RoB OVERALL	Applicability domains (*P*/I/*R*)	APP. OVERALL
**I. Restorative and caries**				
*Baquhaizel et al. (2023)* [19]	U/H/L/L	**High**	H/H/H	**High**
*Yavsan et al. (2025)* [20]	U/L/L/L	**Unclear**	H/H/H	**High**
*Choi et al. (2018)* [21]	U/H/L/L	**High**	L/L/L	**Low**
*Zheng et al. (2021)* [22]	H/L/U/L	**High**	L/L/L	**Low**
*Zhu et al. (2022)* [23]	H/L/L/L	**High**	L/L/L	**Low**
*Altukroni et al. (2023)* [24]	H/L/L/L	**High**	L/L/L	**Low**
*Liu et al. (2024)* [25]	H/L/H/H	**High**	L/L/L	**Low**
*Khan et al. (2025)* [26]	H/U/L/L	**High**	H/L/L	**Low**
*Priya et al. (2024)* [27]	U/L/L/L	**Unclear**	L/L/L	**Low**
*Ying et al. (2024)* [28]	H/L/L/L	**High**	L/L/L	**Low**
*Liu et al. (2025)* [29]	H/L/L/L	**High**	L/L/L	**Low**
**II. Endodontics**				
*Xu et al. (2022)* [30]	H/H/L/H	**High**	H/H/H	**High**
*Chuo et al. (2022)* [31]	U/L/L/L	**Unclear**	H/H/H	**High**
*Ma et al. (2025)* [32]	U/L/L/L	**Unclear**	L/L/L	**Low**
*Pauwels et al. (2021)* [33]	H/L/L/L	**High**	H/L/L	**High**
*Moidu et al. (2022)* [34]	H/L/L/L	**High**	L/H/H	**Low**
*Liu et al. (2025)* [35]	U/U/L/U	**Unclear**	L/L/L	**Low**
*Çetinkaya et al. (2025)* [36]	H/L/L/L	**High**	L/L/L	**Low**
*Çatmabacak and Çetinkaya (2025)* [37]	H/L/L/L	**High**	L/L/L	**Low**
*Ozsari et al. (2025)* [38]	U/L/L/L	**Unclear**	L/L/L	**Low**
*Jin et al. (2025)* [39]	L/L/L/L	**Low**	L/L/L	**Low**
*Dennis et al. (2024)* [40]	H/L/L/L	**High**	L/L/L	**Low**
*Karkehabadi et al. (2024)* [41]	U/L/L/L	**Unclear**	L/L/L	**Low**
**III. Periodontology and implantology**				
*Chen et al. (2023)* [42]	H / U / L / U	**High**	H / H / H	**High**
*Santos et al. (2021)* [43]	H / L / L / L	**High**	L / L / L	**Low**
*Kabir et al. (2021)* [44]	H / L / L / L	**High**	L / L / L	**Low**
*Vera et al. (2023)* [45]	U / L / L / L	**Unclear**	L / L / L	**Low**
*Park et al. (2023)* [46]	L / L / L / L	**Low**	L / L / L	**Low**
*Chen et al. (2024)* [47]	H / L / L / L	**High**	L / H / H	**Low**
*Dai et al. (2024)* [48]	H / L / L / L	**High**	L / L / L	**Low**
*Yavuz et al. (2024)* [49]	H / L / L / H	**High**	L / L / L	**Low**
*Dujic et al. (2023)* [50]	U / L / L / L	**Unclear**	L / L / L	**Low**
*Vilkomir et al. (2024)* [51]	H / L / L / L	**High**	L / L / L	**Low**
*Lin et al. (2024)* [52]	U / L / L / L	**Unclear**	L / L / L	**Low**
*Adnan et al. (2024)* [53]	H / L / L / L	**High**	L / L / L	**Low**
*Fasih et al. (2025)* [54]	H / L / L / L	**High**	L / L / L	**Low**
*Lin et al. (2025)* [55]	L / L / L / L	**Low**	H / H / H	**High**
*Şahin and Eninanç (2025)* [56]	H / L / L / L	**High**	L / H / H	**Low**
**IV. Anatomical detection and workflow**				
*Görürgöz et al. (2022)* [57]	H / L / L / U	**High**	H / H / H	**High**
*Kyventidis and Angelopoulos (2021)* [58]	L / L / H / L	**High**	L / L / L	**Low**
*Mori et al. (2022)* [59]	H / L / L / L	**High**	L / H / H	**Low**
*Pfänder et al. (2023)* [60]	L / L / L / L	**Low**	L / L / L	**Low**
*Ayyıldız et al. (2024)* [61]	U / L / L / L	**Unclear**	L / L / L	**Low**
**V. Multitask AI Models**				
*Chen et al. (2023) *[62]	U / L / L / L	**Unclear**	L / L / L	**Low**
*Khan (2021)* [63]	H / L / L / H	**High**	L / L / L	**Low**
*Chen et al. (2023)* [64]	U / U / L / L	**Unclear**	H / H / H	**High**
*Liu et al. (2024)* [3]	U / U / L / U	**Unclear**	L / L / L	**Low**

The studies are categorized by clinical domain. (*L*: Low risk; *H*: High risk; *U*: Unclear risk; *P*: Patient Selection, *I*: Index Test, *R*: Reference Standard, *F*: Flow and Timing)

### Diagnostic accuracy and clinical performance

**Fig. 3:**
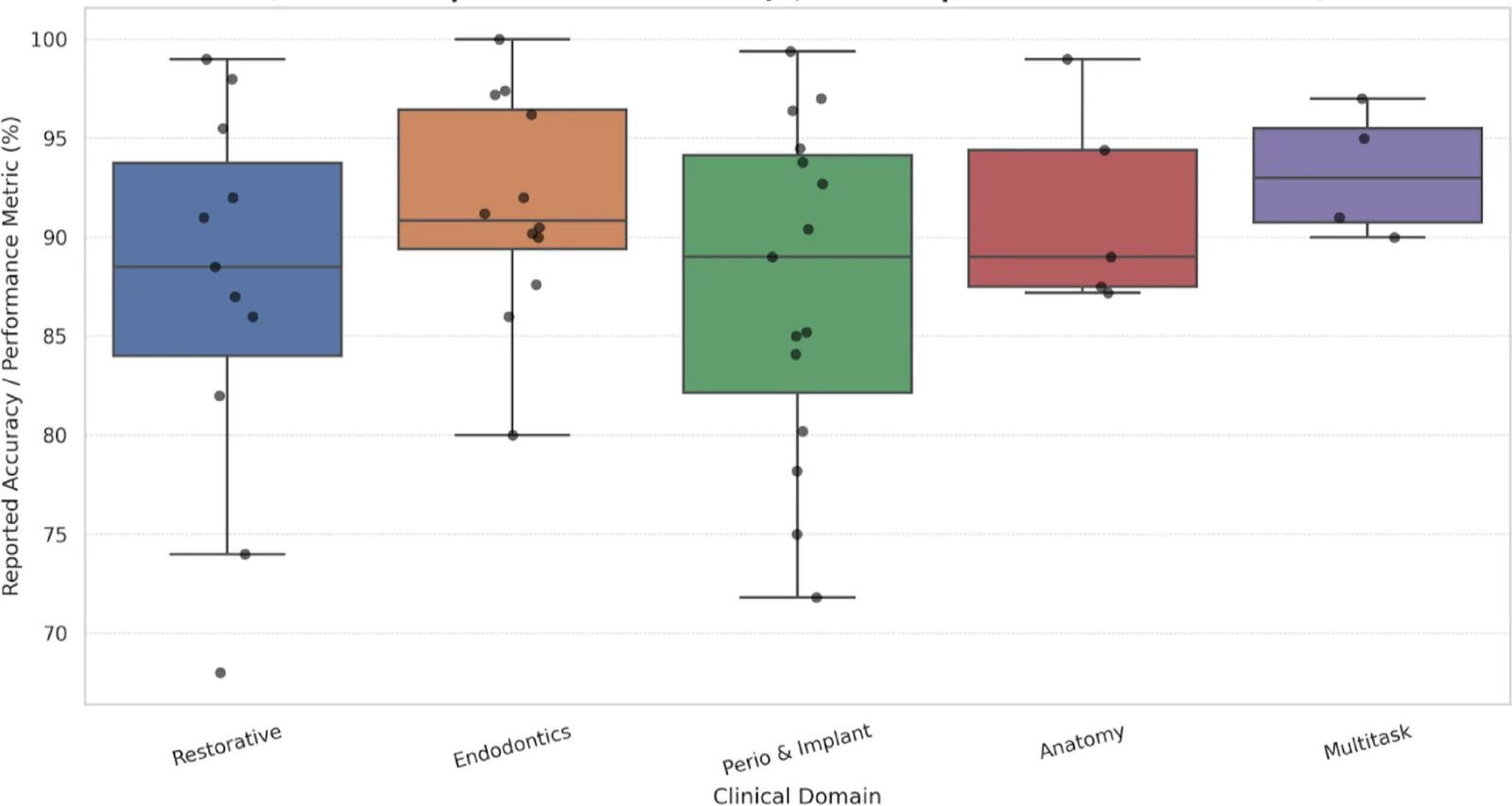
Box plot analysis of diagnostic accuracy across five clinical domains

Due to the significant heterogeneity in study designs, datasets, and performance metrics, a meta-analysis was not feasible. Therefore, a descriptive synthesis of the diagnostic accuracy is presented below, categorized by clinical domain. As detailed in the methodology, a quantitative meta-analysis was not conducted due to significant heterogeneity. Instead, the distribution of diagnostic accuracy across the five clinical domains is visually synthesized in Fig. 3. This box plot analysis illustrates the median performance levels and the interquartile ranges (IQR) for each category, revealing considerable variability within and between the fields. While the ‘Periodontology and Implantology’ domain demonstrates a compact distribution indicative of consistent high performance, the ‘Restorative Dentistry’ domain exhibits a wider range, reflecting the impact of diverse dataset qualities and target lesions.

A critical observation across all clinical domains was the predominant use of pooled datasets for performance evaluation. The vast majority of the included studies reported aggregate diagnostic metrics without stratifying their results by specific anatomical regions (e.g., maxilla versus mandible, or anterior versus posterior teeth). Only a few isolated studies restricted their datasets to highly specific regions, such as focusing exclusively on mandibular molars [51], primary molars [32], or maxillary canines [59]. Furthermore, only one study by Kyventidis and Angelopoulos [58] specifically addressed anatomical region classification. Notably, comparative regional analyses of diagnostic accuracy remain exceedingly rare; a significant exception is the study by Dujic et al. [50], which stratified AI performance across sextants, demonstrating peak accuracy in mandibular anterior teeth and the lowest accuracy in maxillary posterior teeth.

#### Restorative dentistry and caries detection

Studies in this domain (*n* = 11) focused primarily on caries detection, depth classification, and pulp exposure identification. The reported diagnostic accuracy varied widely, ranging from 68% to 99%. Early studies using smaller datasets [21] reported lower performance metrics (F1-Score: 74%). However, recent models utilizing advanced augmentation techniques [19] achieved sensitivity and specificity values exceeding 98%. A key observation in this category is the heavy reliance on cropped Regions of Interest to boost performance, which may not fully reflect the complexity of detecting caries in full-arch periapical radiographs.

#### Endodontic applications

Endodontic AI models (*n* = 12) demonstrated robust performance, particularly in the detection of periapical lesions and broken instruments. The accuracy for these tasks ranged from 80% to 100%, with most recent studies (2024–2025) clustering around the 90–95% accuracy mark. Notably, studies focusing on specific, high-contrast objects like broken files [36] reported exceptionally high precision (> 97%) However, models attempting more subjective tasks, such as assessing the “difficulty” of root canal treatment [41], showed slightly lower performance (Accuracy ~ 87%), highlighting the challenge of quantifying subjective clinical parameters.

#### Periodontology and implantology

This category (*n* = 15) represented the most “technologically mature” domain in periapical radiography. Deep learning models for implant classification and marginal bone loss detection consistently yielded high performance, with accuracy rates frequently exceeding 94%. For instance, implant brand and size classification tasks [46] achieved near-perfect results (> 99%), likely due to the distinct and rigid geometrical features of dental implants compared to biological tissues. Periodontal bone loss detection also showed high stability, although performance dipped slightly when differentiating between specific stages of periodontitis [48].

#### Anatomical detection and multitask models

Models dedicated to anatomical workflow tasks (*n* = 5), such as tooth numbering and radiograph orientation, proved to be highly reliable, with recall rates generally above 90% [57]. A significant emerging trend is the development of Multitask Learning models (*n* = 4). These systems are capable of diagnosing multiple conditions [62] simultaneously with an accuracy comparable to single-task models (~ 95%). This suggests a shift towards comprehensive, “all-in-one” AI diagnostic tools that better mimic the holistic evaluation performed by clinicians.

## Discussion

A substantial portion of the existing literature on dental AI has predominantly focused on panoramic radiography. Panoramic images are frequently favored by deep learning researchers due to their broad anatomical coverage in a single scan and the relative ease of harvesting large-scale, generalized datasets from hospital archives. Consequently, the diagnostic performance of AI on panoramic radiographs has already been extensively synthesized across numerous systematic reviews and meta-analyses, covering broad applications such as caries detection, periodontal bone loss, and anatomical landmark identification [65, 66]. However, despite their widespread utility for general screening, panoramic radiographs suffer from inherent limitations, including geometric distortion, lower spatial resolution, and the superimposition of anatomical structures. These drawbacks render them inadequate for assessing fine morphological details [67]. Recognizing these inherent limitations of panoramic imaging, there is a critical need to evaluate AI applications in modalities that offer superior image quality and localized diagnostic precision. Periapical radiographs, characterized by their high spatial resolution and minimal distortion, remain the gold standard for detecting subtle endodontic and periodontal pathologies. Therefore, this systematic review aims to critically synthesize the current evidence regarding the diagnostic performance of artificial intelligence specifically within periapical radiography, addressing a significant gap in the literature that has historically leaned toward broader, less detailed screening tools. This study provides a highly focused and methodologically rigorous synthesis of how deep learning models navigate the unique challenges of periapical imaging such as limited fields of view and sensor positioning variables.

The selection process of this systematic review was governed by a hierarchical set of exclusion criteria designed to ensure methodological homogeneity and mitigate the inherent risks of AI research, such as overfitting and lack of generalizability. Unlike previous reviews that may include all available literature, we intentionally excluded studies utilizing traditional machine learning algorithms (e.g., SVM, Random Forest) that require manual feature extraction. This decision was made to focus exclusively on Deep Learning (DL) architectures, which are currently at the forefront of dental imaging automation and represent a shift from human-engineered features to autonomous pattern recognition.

To enhance methodological reliability, stringent standards for dataset robustness were implemented in addition to the focus on deep learning. We established a minimum threshold of 200 images grounded in the Central Limit Theorem [13] to ensure that test sets were large enough to provide stable performance estimates and reliable confidence intervals. Studies failing to reach this threshold were excluded to avoid the small sample size trap where validation results often fail to replicate in external clinical environments. Furthermore, the mandatory requirement for data augmentation in small to medium datasets of less than 2000 images reflects a commitment to clinical reality. Without such synthetic variance, deep learning models tend to memorize specific datasets rather than learning the underlying pathology, which is a common pitfall that often leads to inflated accuracy scores in dental AI literature. Finally, we followed the CLAIM criteria to ensure that only the most clinically reliable studies were included. We specifically excluded papers that did not clearly define their test sets or lacked a transparent reference standard, as these issues often lead to data leakage. We also decided to exclude studies that only reported ‘Accuracy’; in unbalanced medical datasets, this metric can be misleading and may hide a model’s inability to actually detect disease. As shown in the PRISMA flow diagram **(**Fig. 1**)**, this filtering process allowed us to identify 47 studies that represent a methodologically strong core of the current literature.

The PRISMA flow diagram presented in Fig. 1 outlines the rigorous selection process employed in this review. Although the initial search yielded a high volume of records (*n* = 660), reflecting the surge in AI research in recent years, the sharp decline to only 47 included studies reveals a critical gap between research quantity and methodological quality. A significant portion of full-text articles (*n* = 69) were excluded, with the majority (*n* = 42) rejected due to fundamental methodological flaws such as the lack of data augmentation or unclear data splitting. This strict exclusion criteria was essential because, as noted in the methodology, training models on small datasets without augmentation leads to overfitting, rendering the reported high accuracy metrics unreliable for external populations. Furthermore, the exclusion of studies with undefined reference standards (*n* = 8) underscores that adherence to reporting guidelines like CLAIM is not yet universal in the field.

Regarding the quality of the included evidence, the QUADAS-2 assessment summarized in Fig. 2 highlights a predominant “High Risk of Bias,” particularly within the “Patient Selection” domain. QUADAS-2 remains the established gold standard for evaluating diagnostic accuracy studies; however, as recent methodological reviews point out, applying its traditional criteria to modern AI models can be challenging, occasionally leading to variable inter-rater agreement and crude categorization of bias without intermediate options [69]. Nevertheless, its rigorous application in our review successfully exposed critical vulnerabilities in the dental AI literature. In practical terms, the extensive red segments in the chart indicate that most studies did not validate their models on a realistic, ‘messy’ representation of the patient population. Instead, researchers frequently utilized retrospective datasets enriched with ‘perfect’ examples of pathologies while excluding ambiguous cases. As demonstrated by Jayakumar et al., the unjustified exclusion of patients—such as deliberately discarding blurry, out-of-focus, or anatomically challenging radiographs—artificially inflates diagnostic accuracy metrics and compromises external validity [70, 71]. This introduces spectrum bias, where the AI is tested on data that is significantly easier to diagnose than what a clinician encounters daily. Consequently, the near-perfect accuracy reported in some restorative studies, such as Baquhaizel et al. [19] who achieved 99% accuracy using VGG16, likely represents an overestimation due to the high risk of bias in patient selection.

Furthermore, a critical methodological limitation across the evaluated literature—and a fundamental characteristic dictated by the retrospective nature of these datasets—is the definition of the ‘gold standard’ or ground truth used to determine diagnostic accuracy. In an ideal experimental design, the absolute ground truth for radiographic pathologies should be established through definitive, multi-modal clinical validation, such as histological examination for periapical lesions, 3D imaging (CBCT) verification, or direct surgical exploration. However, because such absolute ground truths are practically unattainable in large-scale retrospective 2D datasets, all studies included in this review necessarily relied on human expert interpretation (ranging from single-observer annotations to multi-expert consensus panels) as their surrogate reference standard [71].

As emphasized by Willemink et al. [16] and Sounderajah et al. [71], utilizing human interpretation as the ground truth inherently transfers human subjectivity, visual limitations, and cognitive biases directly into the AI algorithm. Consequently, these AI models are fundamentally optimized to replicate human expert opinion rather than to independently discover absolute pathological truths. The quality of this human-derived ground truth also varied significantly. As reflected in our QUADAS-2 assessment, studies relying on a single observer without a robust consensus mechanism or intra-examiner calibration were rated as having a high risk of bias in the Reference Standard domain [25, 30]. Only a minority of studies, such as those by Park et al. [46] and Jin et al. [39] demonstrated a “Low” risk of bias across all domains, setting a necessary benchmark for future clinical reliability.

As visualized in Fig. 3, a quantitative meta-analysis was not conducted because of the extreme statistical and clinical heterogeneity observed across the included studies. The box plots illustrate more than just median accuracy as they also reveal the consistency or the significant dispersion of performance metrics within each clinical domain. For instance, the Periodontology and Implantology domain exhibits the most compact and elevated distribution which indicates high and stable AI performance. This consistency is largely due to the nature of the targets. Unlike diffuse biological pathologies, dental implants and bone levels possess rigid and distinct geometrical boundaries. This clarity allows models to achieve exceptionally high precision, such as the accuracy of over 99% reported for implant size classification by Park et al. [46]. On the other hand, the Restorative Dentistry domain displays a much taller box plot with a wide performance range from 68% to 99%. This variability reflects the complexity of detecting caries on 2D radiographs as well as inconsistent data and varying ground truth definitions. While early studies like Choi et al. [21] appear at the lower end, recent models using data augmentation cluster at the top. However, many high performing studies achieved these results by analyzing cropped regions of interest rather than full radiographs. Given this dispersion, providing a single pooled estimate would be mathematically inaccurate and clinically misleading. In the Endodontics domain, AI excelled at objective tasks such as identifying broken instruments as shown by Çetinkaya et al. [36] with 97.4% accuracy. However, performance was lower in subjective tasks. For example, Karkehabadi et al. [41] reported around 87% accuracy for determining root canal difficulty, suggesting that AI is better at seeing objects than understanding clinical nuance. Finally, the Multitask category represents an evolution toward comprehensive diagnostics. Unlike single task models, multitask architectures mimic a clinician’s holistic workflow. Recent work by Chen et al. [62] and Liu et al. [3] shows these models can diagnose multiple conditions simultaneously with about 95% accuracy. This indicates that the future lies in all in one systems, though these models require rigorous validation on multi center datasets to ensure reliability in general practice.

A notable observation in this literature review is how little research has been dedicated to Pediatric Dentistry. Despite the surge of interest in dental AI, only two studies focused exclusively on children were found. Specifically, Yavsan et al. [20] applied YOLOv8 for detecting approximal caries and Ma et al. [32] used EfficientNet-B2 to classify irreversible pulpitis in primary molars. This lack of data represents a significant oversight in current research. AI models trained on permanent teeth often fail to perform accurately in pediatric cases due to the unique anatomical and physiological differences found in children [68]. Although bitewing radiography is frequently preferred for routine caries screening in children, periapical radiography remains the clinical gold standard for assessing pulpal pathologies, periapical infections, dental trauma, and developmental anomalies in pediatric dentistry [72]. Our investigation suggests that the scarcity of pediatric studies is not solely due to the clinical preference for bitewing radiography in caries screening. Rather, it reflects a broader challenge in dataset acquisition for primary dentition, where the dynamic nature of tooth eruption, root resorption, and the presence of underlying permanent tooth germs create a highly complex ‘background’ for AI algorithms [68]. Therefore, the significant lack of AI studies utilizing pediatric periapical radiographs cannot be entirely explained by imaging modality preferences for caries; rather, it highlights a critical gap in AI development for pediatric endodontic and trauma applications.

Similarly, the diagnostic accuracy of AI models in periapical radiography is not uniform across all anatomical regions, a critical clinical nuance frequently overlooked in the current literature. The distortion and image quality of periapical radiographs vary significantly depending on the tooth location. For instance, the maxillary posterior region frequently suffers from anatomical superimpositions, such as the zygomatic process and the sinus floor, combined with the morphological complexity of multi-rooted teeth. In contrast, mandibular anterior radiographs typically offer clearer visualization with minimal overlapping structures [73]. As highlighted in our results, the widespread reliance on pooled datasets masks these crucial regional variations. When performance is not stratified—as evidenced by the stark contrast reported by Dujic et al. [50] (up to 96.0% accuracy in anterior vs. 78.0% in posterior regions) —a model’s high overall accuracy may be disproportionately driven by its success in distinct anterior regions, thereby concealing significant diagnostic shortcomings in complex posterior areas. Therefore, future studies must prioritize region-specific performance metrics (e.g., anterior vs. posterior, maxilla vs. mandible) to ensure reliable clinical applicability across the entire dental arch.

### Limitations

The substantial heterogeneity in dataset sizes (ranging from approximately 300 to over 10,000 images), variability in ground truth determination methods, and inconsistency in reported performance metrics prevented a reliable quantitative comparison across studies. These methodological discrepancies precluded the conduct of a formal meta-analysis and limited the ability to derive pooled diagnostic accuracy estimates. Furthermore, our methodological appraisal highlighted inherent limitations in the risk-of-bias assessment process itself. The traditional QUADAS-2 tool lacks AI-specific criteria and employs a crude categorization system (lacking an intermediate option), which occasionally led to variable inter-rater agreement and challenges in perfectly stratifying the methodological quality of the included AI studies.

## Conclusions

This systematic review demonstrates that AI models possess a high diagnostic potential in periapical radiography, performing at a level comparable to human experts in tasks such as pathology detection, anatomical segmentation, and implant classification. Although AI offers a revolutionary promise as a ‘second opinion’ in periapical radiology, several critical requirements must be met to solidify its clinical reliability. Specifically, there is an urgent need for more transparent reporting standards, multi-center external validation studies, datasets that accurately reflect real-world clinical prevalence, and the adoption of AI-specific quality assessment frameworks, such as the CLAIM guidelines and the newly proposed QUADAS-AI tool. Ensuring these standards will be essential for mitigating spectrum bias and transitioning AI from experimental success to a dependable tool in routine dental practice.

## Supplementary Information

Below is the link to the electronic supplementary material.

Supplementary Material 1

## Data Availability

The datasets generated and/or analysed during the current study are available from the corresponding author on reasonable request.
