# Supplementary material for: Diagnostic performance of artificial intelligence in periapical radiography: a systematic review
Source: Odontology. 2026 May 13;114(4):1848–64. doi: 10.1007/s10266-026-01401-8 (PMC13630898; doi:10.1007/s10266-026-01401-8)
Supplement: Supplementary file 1 [file 10266_2026_1401_MOESM1_ESM.pdf]

Prerequisites for Publication

Articles other than reviews are accepted for exclusive publication in *Odontology*. By submitting a paper, authors guarantee that they have not submitted and will not submit a similar paper for publication elsewhere in any language. Articles accepted for publication become the property of The Society of The Nippon Dental University and may not be published elsewhere in the same form without the written consent of the Editorial Board.

The editors of *Odontology* subscribe to recommendations formulated by the International Committee of Medical Journal Editors [Ann Intern Med (1988) 108:258– 265] regarding criteria for authorship. Accordingly, each person listed as an author or coauthor for a submitted report (not including review articles) must meet all three of the following criteria.

An author or coauthor must have:

1. C onceived, planned, and performed the work leading to the report, or interpreted the evidence presented, or both.
2. Written the report or reviewed successive versions and shared in their revisions.
3. A pproved the final version.

Meeting these criteria should provide each author with sufficient knowledge of and participation in the work that he or she can accept public respon sibility for the report. The senior or corresponding author is requested to certify that all listed authors meet the above three criteria.

Investigations in humans must be in accordance with the ethical standards of the responsible committee (instit utional or regional) on human ex perimentation or with the Helsinki Declaration of 1964 and all subsequent revisions.

Papers are welcomed from members of the Society and nonmembers alike.

Certification Form — to be submitted with the manuscript

Odontology

Manuscript title: **Diagnostic Performance of Artificial Intelligence in Periapical Radiography: A Systematic Review**

Authors' names: Tulin Tasdemir, Melek Tassoker, Abdullah Arpacı, Ayşenur Kula, Ferhat Enes Kocabiyik, Mehmet Enes İyişenyürek, Huseyin Hakli

I hereby certify that the authors of the above manuscript have all:
1. C onceived, planned, and performed the work leading to the report, or interpreted the evidence presented, or both;
2. W ritten the report or reviewed successive versions and shared in their revisions; and
3. A pproved the final version.

Further, I certify that:

1. This work has not been published elsewhere;
2. Humane procedures have been followed in the treatment of exp erimental animals; and
3. This paper has been carefully read by a native English speaker who is familiar with the field of work (this applies to authors who are not fluent in English).

Signature: ______________________ Date: 03.02.2026
